# (2*E*)-2-(3,4-Di­meth­oxy­benzyl­idene)-3,4-di­hydro­naphthalen-1(2*H*)-one

**DOI:** 10.1107/S2414314621003096

**Published:** 2021-03-26

**Authors:** D. Angeline Shirmila, D. Reuben Jonathan, M. Krishna Priya, J. Hemalatha, G. Usha

**Affiliations:** a PG and Research Department of Physics, Queen Mary’s College, Affiliated to University of Madras, Chennai-4, Tamil Nadu, India; b Department of Chemistry, Madras Christian College, Affiliated to University of Madras, Chennai-59, Tamil Nadu, India; University of Aberdeen, Scotland

**Keywords:** crystal structure, chalcone derivative

## Abstract

In the title chalcone derivative, C_19_H_18_O_3_, the cyclo­hexa­none ring adopts a distorted half-chair conformation and the dihedral angle between the aromatic rings is 52.20 (15)°. In the crystal, weak C—H⋯O hydrogen bonds link the mol­ecules into *C*(12) [001] chains.

## Abstract 2

In the title chalcone derivative, the cyclo­hexa­none ring adopts a distorted half-chair conformation and the dihedral angle between the aromatic rings is 52.20 (15)°. In the crystal, weak C—H⋯O hydrogen bonds link the mol­ecules into *C*(12) [001] chains.

## Graphical abstract

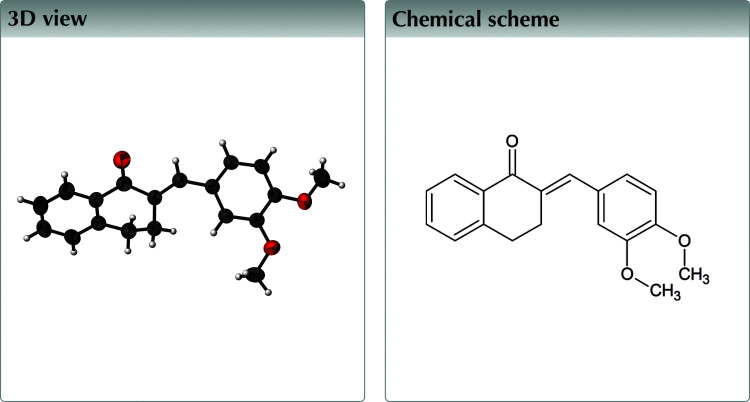

## Structure description

**Figure 1:**
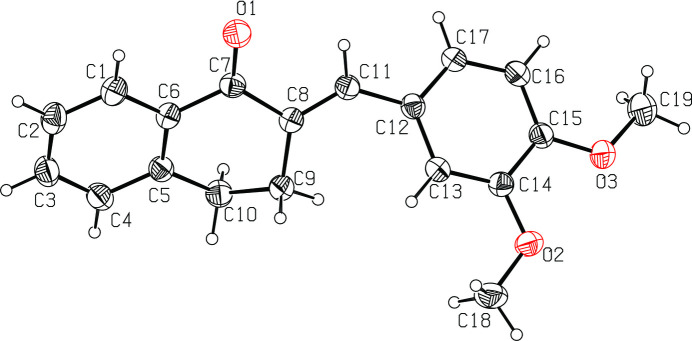
The mol­ecular structure of the title compound, showing the atom labelling. Displacement ellipsoids are drawn at 30% probability level. Hydrogen atoms are shown as arbitrary spheres.

Chalcone derivatives exhibit various biological activities (Tajuddeen *et al.*, 2018 ▸) and those that crystallize in non-centrosymmetric space groups are candidates for non-linear optical materials (Shettigar *et al.*, 2006 ▸). As part of our studies in this area, we now describe the synthesis and structure of the title compound, C_19_H_18_O_3_, (I), (Fig. 1 ▸).

The geometrical data for (I) are similar to those in related structures (Biruntha *et al.*, 2018 ▸; Baydere *et al.*, 2019 ▸). The C5–C10 cyclo­hexa­none ring adopts a distorted half-chair conformation with C9 and C10 deviating from C5–C8 (r.m.s. deviation = 0.086 Å) by −0.381 (3) and 0.285 (4) Å, respectively. The dihedral angle between the C1–C6 and C12–C17 aromatic rings is 52.20 (15)° and the C atoms of both meth­oxy groups lie close to the C12–C17 plane [deviations = 0.057 (4) for C18 and 0.148 (6) Å for C19].

**Figure 2:**
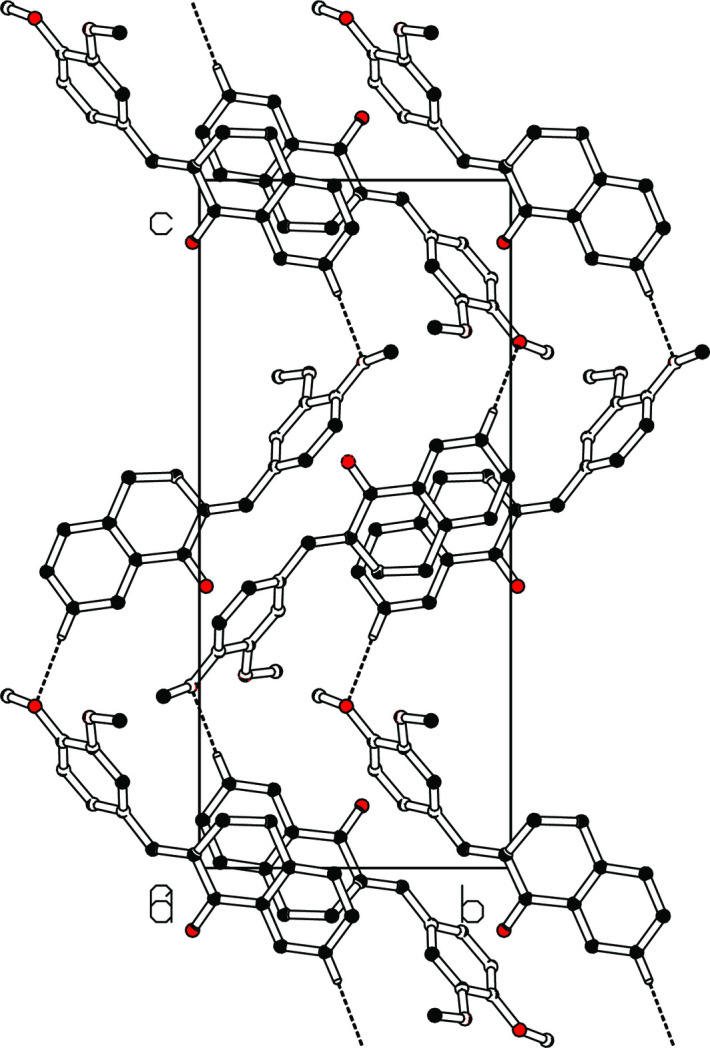
Packing of the mol­ecules along the *c* axis with C—H⋯O inter­actions running in a zigzag head-to-tail fashion, viewed along the *a* axis. The hydrogen bonds are shown as dashed lines. Hydrogen atoms not involved in hydrogen bonds are omitted for clarity.

In the crystal, weak C2—H2⋯O3 hydrogen bonds link the mol­ecules into *C*(12) zigzag chains propagating in the [001] direction with adjacent mol­ecules related by a 2_1_ screw axis (Table 1 ▸, Fig. 2 ▸). The chains pack without any identifiable directional inter­actions between them beyond van der Waals contacts.

## Synthesis and crystallization

The title compound was prepared by a Claisen–Schmidt condensation (Dong *et al.*, 2008 ▸): equimolar qu­anti­ties of 3,4-dimeth­oxy benzaldehyde (2.51 g, 0.015 mol) and α-tetra­lone (2.0 ml, 0.015 mol) were dissolved in ethanol in a 250 ml conical flask and stirred for 15 min. Freshly prepared 10% NaOH solution was added to the mixture and stirred again for 1 h. This mixture was kept at room temperature for 24 h and then poured into ice-cold water. A yellow precipitate formed, which was washed with distilled water to remove any traces of NaOH. The filtered, dried crude product was recrystallized three times from acetone solution. After four days, yellow blocks of (I) were harvested (yield 78%; m.p. 110°C).

## Refinement

Crystal data, data collection and structure refinement details are summarized in Table 2 ▸.

## Supplementary Material

Crystal structure: contains datablock(s) I. DOI: 10.1107/S2414314621003096/hb4379sup1.cif

Structure factors: contains datablock(s) I. DOI: 10.1107/S2414314621003096/hb4379Isup2.hkl

Supporting information file. DOI: 10.1107/S2414314621003096/hb4379Isup3.cml

CCDC reference: 1968703

Additional supporting information:  crystallographic information; 3D view; checkCIF report

## Figures and Tables

**Table 1:** Hydrogen-bond geometry (Å, °)

*D*—H⋯*A*	*D*—H	H⋯*A*	*D*⋯*A*	*D*—H⋯*A*
C2—H2⋯O3^i^	0.93	2.52	3.385 (4)	155

Symmetry code: (i) 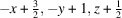 .

**Table 2:** Experimental details

Crystal data	
Chemical formula	C_19_H_18_O_3_
*M*_r_	294.33
Crystal system, space group	Orthorhombic, *P*2_1_2_1_2_1_
Temperature (K)	296
*a*, *b*, *c* (Å)	7.9229 (15), 9.4474 (19), 20.875 (4)
*V* (Å^3^)	1562.5 (5)
*Z*	4
Radiation type	Mo *K*α
μ (mm^−1^)	0.08
Crystal size (mm)	0.20 × 0.15 × 0.15

Data collection	
Diffractometer	Bruker Kappa *APEX3* CMOS
Absorption correction	Multi-scan (*SADABS*; Bruker 2016 ▸)
*T*_min_, *T*_max_	0.985, 0.987
No. of measured, independent and observed [*I* > 2σ(*I*)] reflections	34283, 3886, 3101
*R*_int_	0.043
(sin θ/λ)_max_ (Å^−1^)	0.667

Refinement	
*R*[*F* ^2^ > 2σ(*F* ^2^)], *wR*(*F* ^2^), *S*	0.057, 0.180, 1.04
No. of reflections	3886
No. of parameters	200
H-atom treatment	H-atom parameters constrained
Δρ_max_, Δρ_min_ (e Å^−3^)	0.24, −0.23
Absolute structure	Flack *x* determined using 1195 quotients [(*I* ^+^)−(*I* ^−^)]/[(*I* ^+^)+(*I* ^−^)] (Parsons *et al.*, 2013 ▸)
Absolute structure parameter	0.5 (3)

Computer programs: *APEX3* and *SAINT* (Bruker, 2016 ▸), *SHELXT2014/4* (Sheldrick, 2015*a* ▸), *SHELXL2018/3* (Sheldrick, 2015*b* ▸) and *ORTEP-3 for Windows* (Farrugia, 2012 ▸).

## full crystallographic data

### Crystal data

**Table:**

C_19_H_18_O_3_	*D*_x_ = 1.251 Mg m^−^^3^
*M**_r_* = 294.33	Mo *K*α radiation, λ = 0.71073 Å
Orthorhombic, *P*2_1_2_1_2_1_	Cell parameters from 9801 reflections
*a* = 7.9229 (15) Å	θ = 2.9–28.2°
*b* = 9.4474 (19) Å	µ = 0.08 mm^−^^1^
*c* = 20.875 (4) Å	*T* = 296 K
*V* = 1562.5 (5) Å^3^	Block, yellow
*Z* = 4	0.20 × 0.15 × 0.15 mm
*F*(000) = 624	

### Data collection

**Table:**

Bruker Kappa APEX3 CMOS diffractometer	3101 reflections with *I* > 2σ(*I*)
Radiation source: fine-focus sealed tube	*R*_int_ = 0.043
ω and φ scan	θ_max_ = 28.3°, θ_min_ = 3.4°
Absorption correction: multi-scan (SADABS; Bruker 2016)	*h* = −10→10
*T*_min_ = 0.985, *T*_max_ = 0.987	*k* = −12→12
34283 measured reflections	*l* = −22→27
3886 independent reflections	

### Refinement

**Table:**

Refinement on *F*^2^	H-atom parameters constrained
Least-squares matrix: full	*w* = 1/[σ^2^(*F*_o_^2^) + (0.1074*P*)^2^ + 0.3866*P*] where *P* = (*F*_o_^2^ + 2*F*_c_^2^)/3
*R*[*F*^2^ > 2σ(*F*^2^)] = 0.057	(Δ/σ)_max_ < 0.001
*wR*(*F*^2^) = 0.180	Δρ_max_ = 0.24 e Å^−^^3^
*S* = 1.04	Δρ_min_ = −0.23 e Å^−^^3^
3886 reflections	Extinction correction: SHELXL2018/3 (Sheldrick 2015b), Fc^*^=kFc[1+0.001xFc^2^λ^3^/sin(2θ)]^-1/4^
200 parameters	Extinction coefficient: 0.35 (3)
0 restraints	Absolute structure: Flack *x* determined using 1195 quotients [(*I*^+^)-(*I*^-^)]/[(*I*^+^)+(*I*^-^)] (Parsons *et al.*, 2013)
Hydrogen site location: inferred from neighbouring sites	Absolute structure parameter: 0.5 (3)

### Special details

**Table:**

Geometry. All esds (except the esd in the dihedral angle between two l.s. planes) are estimated using the full covariance matrix. The cell esds are taken into account individually in the estimation of esds in distances, angles and torsion angles; correlations between esds in cell parameters are only used when they are defined by crystal symmetry. An approximate (isotropic) treatment of cell esds is used for estimating esds involving l.s. planes.
Refinement. Hydrogen atoms were fixed geometrically and treated as riding atoms, with C—H = 0.93–0.97 Å and *U*_iso_(H)= 1.2*U*_eq_(C) or 1.5*U*_eq_(*C*-methyl).

### Fractional atomic coordinates and isotropic or equivalent isotropic displacement parameters (Å^2^)

**Table:**

	*x*	*y*	*z*	*U*_iso_*/*U*_eq_	
O2	0.6008 (3)	0.1479 (3)	0.28091 (12)	0.0624 (7)	
O3	0.8450 (3)	−0.0282 (3)	0.26479 (12)	0.0649 (7)	
O1	0.9071 (4)	0.4782 (3)	0.59032 (11)	0.0649 (7)	
C13	0.7334 (4)	0.2526 (3)	0.37495 (14)	0.0458 (6)	
H13	0.644379	0.315256	0.381066	0.055*	
C14	0.7278 (4)	0.1574 (3)	0.32452 (14)	0.0464 (7)	
C16	0.9976 (4)	0.0639 (3)	0.35657 (16)	0.0519 (7)	
H16	1.087221	0.001746	0.350367	0.062*	
C15	0.8614 (4)	0.0615 (3)	0.31555 (14)	0.0479 (7)	
C8	0.8296 (4)	0.4838 (3)	0.48040 (13)	0.0441 (6)	
C17	1.0009 (4)	0.1591 (3)	0.40715 (15)	0.0503 (7)	
H17	1.092160	0.158070	0.435149	0.060*	
C6	0.8282 (4)	0.7030 (3)	0.55210 (13)	0.0446 (6)	
C12	0.8722 (4)	0.2556 (3)	0.41712 (14)	0.0449 (6)	
C5	0.8007 (4)	0.7892 (3)	0.49871 (15)	0.0475 (7)	
C11	0.8832 (4)	0.3505 (3)	0.47233 (14)	0.0471 (7)	
H11	0.936499	0.311604	0.507916	0.057*	
C7	0.8610 (4)	0.5492 (3)	0.54443 (13)	0.0467 (7)	
C10	0.8113 (5)	0.7267 (3)	0.43275 (15)	0.0533 (7)	
H10A	0.927775	0.728510	0.418478	0.064*	
H10B	0.745607	0.784317	0.403441	0.064*	
C9	0.7464 (4)	0.5751 (3)	0.43063 (15)	0.0510 (7)	
H9A	0.625376	0.575024	0.437596	0.061*	
H9B	0.767737	0.535552	0.388515	0.061*	
C1	0.8256 (4)	0.7620 (4)	0.61349 (16)	0.0545 (8)	
H1	0.844547	0.704442	0.648899	0.065*	
C4	0.7687 (4)	0.9330 (4)	0.50842 (18)	0.0566 (8)	
H4	0.748799	0.991456	0.473385	0.068*	
C2	0.7953 (5)	0.9042 (4)	0.62230 (18)	0.0616 (9)	
H2	0.794300	0.942688	0.663314	0.074*	
C3	0.7665 (5)	0.9893 (4)	0.5695 (2)	0.0636 (9)	
H3	0.745332	1.085375	0.575098	0.076*	
C18	0.4641 (5)	0.2440 (5)	0.2858 (2)	0.0755 (12)	
H18A	0.384295	0.225412	0.252249	0.113*	
H18B	0.410051	0.232675	0.326635	0.113*	
H18C	0.505437	0.339161	0.281840	0.113*	
C19	0.9838 (8)	−0.1150 (5)	0.2497 (3)	0.0900 (15)	
H19A	0.956607	−0.172841	0.213369	0.135*	
H19B	1.079682	−0.056856	0.239872	0.135*	
H19C	1.009656	−0.174440	0.285713	0.135*	

### Atomic displacement parameters (Å^2^)

**Table:**

	*U*^11^	*U*^22^	*U*^33^	*U*^12^	*U*^13^	*U*^23^
O2	0.0552 (13)	0.0737 (15)	0.0584 (13)	0.0130 (12)	−0.0132 (11)	−0.0127 (12)
O3	0.0695 (15)	0.0658 (14)	0.0595 (13)	0.0162 (13)	−0.0077 (12)	−0.0208 (11)
O1	0.0899 (18)	0.0574 (13)	0.0472 (12)	0.0076 (13)	−0.0097 (12)	0.0031 (10)
C13	0.0449 (14)	0.0442 (13)	0.0484 (15)	0.0035 (12)	0.0014 (11)	0.0008 (12)
C14	0.0465 (15)	0.0472 (15)	0.0454 (14)	0.0009 (12)	−0.0005 (12)	0.0013 (12)
C16	0.0543 (17)	0.0452 (15)	0.0563 (17)	0.0087 (13)	−0.0016 (14)	0.0000 (13)
C15	0.0529 (16)	0.0449 (14)	0.0459 (14)	0.0044 (13)	0.0002 (13)	−0.0013 (11)
C8	0.0437 (14)	0.0459 (14)	0.0426 (13)	−0.0015 (11)	0.0025 (11)	−0.0006 (11)
C17	0.0541 (16)	0.0430 (14)	0.0539 (16)	0.0053 (13)	−0.0081 (14)	0.0004 (13)
C6	0.0434 (14)	0.0482 (14)	0.0423 (14)	−0.0036 (12)	0.0044 (11)	−0.0030 (11)
C12	0.0480 (14)	0.0404 (13)	0.0464 (14)	−0.0012 (12)	0.0010 (12)	0.0017 (11)
C5	0.0449 (14)	0.0449 (14)	0.0526 (16)	−0.0019 (12)	0.0001 (13)	0.0000 (12)
C11	0.0514 (15)	0.0444 (14)	0.0455 (14)	0.0000 (13)	−0.0021 (12)	0.0013 (11)
C7	0.0485 (15)	0.0494 (15)	0.0422 (14)	0.0006 (13)	0.0013 (12)	0.0038 (11)
C10	0.0654 (19)	0.0499 (15)	0.0447 (15)	0.0008 (15)	0.0005 (14)	0.0048 (12)
C9	0.0573 (17)	0.0487 (15)	0.0468 (15)	0.0033 (14)	−0.0040 (14)	−0.0023 (13)
C1	0.0555 (17)	0.0594 (18)	0.0485 (16)	−0.0067 (15)	0.0053 (14)	−0.0046 (14)
C4	0.0534 (16)	0.0466 (15)	0.070 (2)	0.0028 (14)	−0.0008 (15)	0.0021 (15)
C2	0.060 (2)	0.063 (2)	0.062 (2)	−0.0014 (16)	0.0079 (16)	−0.0173 (16)
C3	0.0573 (18)	0.0519 (17)	0.082 (2)	0.0048 (15)	0.0002 (18)	−0.0172 (17)
C18	0.059 (2)	0.094 (3)	0.074 (2)	0.026 (2)	−0.0121 (18)	−0.008 (2)
C19	0.093 (3)	0.095 (3)	0.082 (3)	0.033 (3)	−0.007 (2)	−0.036 (2)

### Geometric parameters (Å, º)

**Table:**

O2—C14	1.360 (4)	C5—C4	1.397 (4)
O2—C18	1.417 (4)	C5—C10	1.500 (4)
O3—C15	1.363 (4)	C11—H11	0.9300
O3—C19	1.407 (5)	C10—C9	1.523 (4)
O1—C7	1.225 (4)	C10—H10A	0.9700
C13—C14	1.385 (4)	C10—H10B	0.9700
C13—C12	1.409 (4)	C9—H9A	0.9700
C13—H13	0.9300	C9—H9B	0.9700
C14—C15	1.406 (4)	C1—C2	1.377 (5)
C16—C15	1.378 (5)	C1—H1	0.9300
C16—C17	1.388 (4)	C4—C3	1.381 (5)
C16—H16	0.9300	C4—H4	0.9300
C8—C11	1.340 (4)	C2—C3	1.384 (6)
C8—C7	1.493 (4)	C2—H2	0.9300
C8—C9	1.502 (4)	C3—H3	0.9300
C17—C12	1.383 (4)	C18—H18A	0.9600
C17—H17	0.9300	C18—H18B	0.9600
C6—C5	1.397 (4)	C18—H18C	0.9600
C6—C1	1.398 (4)	C19—H19A	0.9600
C6—C7	1.485 (4)	C19—H19B	0.9600
C12—C11	1.463 (4)	C19—H19C	0.9600
			
C14—O2—C18	118.3 (3)	C5—C10—H10A	109.2
C15—O3—C19	117.5 (3)	C9—C10—H10A	109.2
C14—C13—C12	120.9 (3)	C5—C10—H10B	109.2
C14—C13—H13	119.6	C9—C10—H10B	109.2
C12—C13—H13	119.6	H10A—C10—H10B	107.9
O2—C14—C13	125.1 (3)	C8—C9—C10	111.8 (3)
O2—C14—C15	115.2 (3)	C8—C9—H9A	109.2
C13—C14—C15	119.7 (3)	C10—C9—H9A	109.2
C15—C16—C17	119.9 (3)	C8—C9—H9B	109.2
C15—C16—H16	120.1	C10—C9—H9B	109.2
C17—C16—H16	120.1	H9A—C9—H9B	107.9
O3—C15—C16	124.6 (3)	C2—C1—C6	121.0 (3)
O3—C15—C14	115.6 (3)	C2—C1—H1	119.5
C16—C15—C14	119.8 (3)	C6—C1—H1	119.5
C11—C8—C7	116.6 (3)	C3—C4—C5	120.7 (3)
C11—C8—C9	126.3 (3)	C3—C4—H4	119.6
C7—C8—C9	117.0 (3)	C5—C4—H4	119.6
C12—C17—C16	121.9 (3)	C1—C2—C3	119.3 (3)
C12—C17—H17	119.1	C1—C2—H2	120.4
C16—C17—H17	119.1	C3—C2—H2	120.4
C5—C6—C1	119.8 (3)	C4—C3—C2	120.6 (3)
C5—C6—C7	120.8 (2)	C4—C3—H3	119.7
C1—C6—C7	119.5 (3)	C2—C3—H3	119.7
C17—C12—C13	117.9 (3)	O2—C18—H18A	109.5
C17—C12—C11	118.6 (3)	O2—C18—H18B	109.5
C13—C12—C11	123.5 (3)	H18A—C18—H18B	109.5
C4—C5—C6	118.6 (3)	O2—C18—H18C	109.5
C4—C5—C10	121.7 (3)	H18A—C18—H18C	109.5
C6—C5—C10	119.6 (3)	H18B—C18—H18C	109.5
C8—C11—C12	131.0 (3)	O3—C19—H19A	109.5
C8—C11—H11	114.5	O3—C19—H19B	109.5
C12—C11—H11	114.5	H19A—C19—H19B	109.5
O1—C7—C6	120.2 (3)	O3—C19—H19C	109.5
O1—C7—C8	121.5 (3)	H19A—C19—H19C	109.5
C6—C7—C8	118.2 (2)	H19B—C19—H19C	109.5
C5—C10—C9	112.2 (3)		
			
C18—O2—C14—C13	1.5 (5)	C17—C12—C11—C8	147.4 (3)
C18—O2—C14—C15	−178.2 (3)	C13—C12—C11—C8	−35.7 (5)
C12—C13—C14—O2	−179.0 (3)	C5—C6—C7—O1	170.2 (3)
C12—C13—C14—C15	0.7 (4)	C1—C6—C7—O1	−9.5 (5)
C19—O3—C15—C16	−6.3 (5)	C5—C6—C7—C8	−12.2 (4)
C19—O3—C15—C14	173.1 (4)	C1—C6—C7—C8	168.1 (3)
C17—C16—C15—O3	−179.5 (3)	C11—C8—C7—O1	−11.9 (5)
C17—C16—C15—C14	1.1 (5)	C9—C8—C7—O1	170.1 (3)
O2—C14—C15—O3	−0.4 (4)	C11—C8—C7—C6	170.6 (3)
C13—C14—C15—O3	179.9 (3)	C9—C8—C7—C6	−7.4 (4)
O2—C14—C15—C16	179.0 (3)	C4—C5—C10—C9	−146.9 (3)
C13—C14—C15—C16	−0.7 (5)	C6—C5—C10—C9	35.0 (4)
C15—C16—C17—C12	−1.6 (5)	C11—C8—C9—C10	−138.6 (3)
C16—C17—C12—C13	1.6 (5)	C7—C8—C9—C10	39.2 (4)
C16—C17—C12—C11	178.7 (3)	C5—C10—C9—C8	−52.3 (4)
C14—C13—C12—C17	−1.1 (4)	C5—C6—C1—C2	0.4 (5)
C14—C13—C12—C11	−178.1 (3)	C7—C6—C1—C2	−179.9 (3)
C1—C6—C5—C4	−0.9 (5)	C6—C5—C4—C3	0.8 (5)
C7—C6—C5—C4	179.4 (3)	C10—C5—C4—C3	−177.3 (3)
C1—C6—C5—C10	177.3 (3)	C6—C1—C2—C3	0.3 (6)
C7—C6—C5—C10	−2.4 (4)	C5—C4—C3—C2	−0.2 (5)
C7—C8—C11—C12	179.4 (3)	C1—C2—C3—C4	−0.4 (6)
C9—C8—C11—C12	−2.8 (6)		

### Hydrogen-bond geometry (Å, º)

**Table:**

*D*—H···*A*	*D*—H	H···*A*	*D*···*A*	*D*—H···*A*
C2—H2···O3^i^	0.93	2.52	3.385 (4)	155

Symmetry code: (i) −*x*+3/2, −*y*+1, *z*+1/2.
